# A Review of Explicit and Implicit Assumptions When Providing Personalized Feedback Based on Self-Report EMA Data

**DOI:** 10.3389/fpsyg.2021.764526

**Published:** 2021-12-08

**Authors:** IJsbrand Leertouwer, Angélique O. J. Cramer, Jeroen K. Vermunt, Noémi K. Schuurman

**Affiliations:** ^1^Department of Methodology and Statistics, Tilburg University, Tilburg, Netherlands; ^2^Department of Methodology and Statistics, Utrecht University, Utrecht, Netherlands

**Keywords:** ecological momentary assessment, retrospective assessment, personalized feedback, insight, intervention, experiencing self, remembering self

## Abstract

Ecological Momentary Assessment (EMA) in which participants report on their moment-to-moment experiences in their natural environment, is a hot topic. An emerging field in clinical psychology based on either EMA, or what we term *Ecological Retrospective Assessment* (ERA) as it requires retrospectivity, is the field of *personalized feedback*. In this field, EMA/ERA-data-driven summaries are presented to participants with the goal of promoting their insight in their experiences. Underlying this procedure are some fundamental assumptions about (i) the relation between true moment-to-moment experiences and retrospective evaluations of those experiences, (ii) the translation of these experiences and evaluations to different types of data, (iii) the comparison of these different types of data, and (iv) the impact of a summary of moment-to-moment experiences on retrospective evaluations of those experiences. We argue that these assumptions deserve further exploration, in order to create a strong evidence-based foundation for the personalized feedback procedure.

## Introduction

Technological advancements in the domain of smartphones have gone hand in hand with increased attention for moment-to-moment experiences of the individual (Hamaker and Wichers, 2017), measured through *ecological momentary assessment* (EMA). In an EMA data collection effort, participants are measured multiple times per day in their natural environment. A prominent emerging field in clinical psychology for which EMA data are used is the field of *personalized feedback*. In a personalized feedback procedure involving *self-report* measures,^1^ participants first partake in a period of EMA, and afterwards receive a summary of their EMA data, for example about the mean of their positive affect in different environments (Kramer et al., 2014; van Roekel et al., 2017; Ornée et al., 2021). The goal of such a procedure is to promote peoples’ insight into, and self-management of, their psychological functioning (Wichers et al., 2011; van Os et al., 2017; Bos et al., 2019).

Underlying this procedure are some fundamental explicit and implicit assumptions, many of which have to do with the presumed merits of EMA. Perhaps most fundamentally, as the goal is to promote insight, the procedure is based on the presumption that peoples’ moment-to-moment experiences, measured with EMA, are different from what they remember themselves about those experiences. That is, only in this case can a summary of their moment-to-moment experiences promote insight.

Our aim is to make such underlying assumptions explicit, so that they may be debated and investigated further in future studies. To this end, we will derive the assumptions in the procedure of providing people with personalized feedback based on EMA, and express them in simple expressions. This practice promotes clarity and helps to establish testable propositions, as recently emphasized by other authors (Haslbeck et al., 2019). We will evaluate the assumptions based on a review of the current empirical literature. Making these underlying assumptions explicit and studying them is essential for disentangling and understanding the processes under the hood of the personalized feedback procedure.

Specifically, we will formalize and evaluate four main assumptions in the personalized feedback procedure. The first three assumptions concern the more general question of how true experiences that people have relate to different measurement procedures of EMA and retrospective assessment. In section “Experiencing and Evaluating Experiences,” we will discuss the first assumption, which is about the core theoretical constructs behind the personalized feedback procedure. This assumption is that people systematically do not represent all their true moment-to-moment experiences in true retrospective evaluations of those experiences. In section “Measuring Experiences in Clinical Research,” we will discuss the second assumption, which is about how the core theoretical constructs are translated to data. The assumption here is that EMA represents true moment-to-moment experiences well, while retrospective assessment does not, as it represents evaluations of moment-to-moment experiences. In section “A Difference Between Measurement Types?,” we will discuss the main assumption in studies that compare different data collection procedures. In these studies, it is often reported that EMA and retrospective assessment do not align, which is generally assumed to be supporting evidence for the notion that true moment-to-moment experiences are different from evaluations of these experiences.

In section “Affecting Evaluations, Using Feedback About Experiences,” we will discuss the fourth main assumption, which pertains to the specific personalized feedback procedure. The procedure can be characterized as an instrumentalization of a difference between moment-to-moment experiences and evaluations of those experiences. That is, in the personalized feedback procedure, it is assumed that personalized feedback based on EMA (i.e., a summary of moment-to-moment experiences) may influence peoples’ retrospective evaluation of their experiences. The specific main assumptions, sub-assumptions, and expressions can be found in Table 1.

**Table 1:** Assumptions, sub-assumptions and expressions evaluated in this manuscript.

	Main assumption		Sub-assumptions	Expressions
1.	People systematically do not represent all their true moment-to-moment experiences in true retrospective evaluations of those experiences	1	Remembered experiences are an unrepresentative incomplete subset of the experiencing self	rememberedexperiences⊂experiencingself
		2	The remembering self applies a summary function to remembered experiences	$rememberingself=default\left(rememberedexperiences\right)$
2.	RA does not represent moment-to-moment experiences well; EMA/ERA represents moment-tot-moment experiences well	5	RA requires a specific summary function over remembered experiences	$RA=formulateRA\left(rememberedexperiencestimeframe\right)$
		6	EMA takes a representative incomplete subset of the experiencing self	EMA⊂experiencingself
3.	EMA, ERA and RA will not align, because of a difference between true moment-to-moment experiences and true retrospective evaluations of those experiences	7	RA and EMA will not align	$formulateRA\left(rememberedexperiencestimeframe\right)\neq aggregateEMA\left(EMA\right)$
		8	ERA and EMA will not align	$aggregateERA\left(formulateERA\left(rememberedexperiencestimeframeshort\right)\right)\neq aggregateEMA\left(EMA\right)$
		9	RA and ERA will not align	$formulateRA\left(rememberedexperiencestimeframe\right)\neq aggregateERA\left(formulateERA\left(rememberedexperiencestimeframeshort\right)\right)$
4.	Personalized feedback based on moment-to-moment experiences can affect retrospective evaluations of those experiences	10	A relevant summary of EMA/ERA-data may change the remembering self, to be more similar to an ideal summary applied to a representative subset of experiences	$feedback\left(EMA\;or\;ERA\right)\to default\left(rememberedexperiences\;pre\right)\approx ideal\left(rememberedexperiencespost\right)$

We will end with a discussion where we summarize evidence for the validity of these assumptions and provide directions for future research. We believe that all of these assumptions require further investigation, in order to ensure that the personalized feedback procedure is based on a relevant difference between representative data sources that actually increases participants’ insight. As the goal of this procedure is to help vulnerable individuals gain insight into their condition, we believe that it is of the utmost importance that we are sure about the methods that should lead to this insight.

## Experiencing and Evaluating Experiences

In the personalized feedback procedure, it is presumed that participants’ moment-to-moment experiences differ from retrospective evaluations of those experiences. That is, if participants would be fully aware of all their moment-to-moment experiences, they would not gain insight from a summary of those experiences. This theoretical distinction between the true process of experiencing and the true process of retrospectively evaluating those experiences will form the start our exploration of assumptions. A true experience in this context can be defined as “*something personally encountered, undergone or lived through*” (Experience, n.d.). By this definition, it is clear that the average individual goes through so many experiences that they will not keep track of all of them. The individual will however be able to retrieve some experiences, in the form of recollections of those experiences. The process of retrieving and evaluating true experiences can be formalized as the true process of retrospective evaluation. Previous authors have suggested that these processes are reflected by different aspects of the self (Kahneman and Riis, 2005; Kahneman, 2011). Kahneman and Riis (2005) propose that a person’s *experiencing self* lives approximately 20.000 experiences per day and barely has time to exist because these experiences are so short, while the *remembering self* is more stable and permanent, as it retrieves and evaluates experiences, and keeps score. Kahneman and colleagues assume that in this process, the remembering self provides a systematically incomplete picture of the experiencing self. This is the first main assumption that we will evaluate (see assumption 1 in Table 1). In the psychological literature, such systematic divergence from the experiencing self is commonly referred to as *retrospective*- or *recall bias* (c.f., Stone and Shiffman, 1994; Shiffman et al., 2008).

The distinction between true experiences and what is remembered about them is an important underlying assumption in the personalized feedback process. In the personalized feedback procedure, this distinction is left implicit in the form of an expectation that there will be a difference between the two, leaving room for interpretation of why and how they may be different. We will make this distinction explicit by specifying the assumption in two simple expressions. In these expressions, we will adopt the *experiencing self* and *remembering self* terminology of Kahneman and colleagues, with a slight transformation. That is, we will refer to the set of all true moment-to-moment experiences as the experiencing self, and will refer to retrospective evaluation in the form of a summary of those experiences as the remembering self. The transformation thus lies in the fact that we will define the experiencing self and remembering self as their end-product (i.e., a set of experiences, and a summary of those experiences, respectively), rather than as actors providing this end-product. Kahneman and Riis (2005) state that the remembering self depends on retrieval and reasonable integration of experiences spread over time. We have reduced these theories about the relationship between the experiencing self and remembering self to the following expressions:


rememberedexperiences⊂experiencingself (1)

$$rememberingself=default\left(rememberedexperiences\right)$$ (2)

Expression 1 specifies the retrieval process, in which the sign signifies that remembered experiences are an *incomplete subset* of all true moment-to-moment experiences of the experiencing self. This subset is assumed to be non-random, as we will address in detail later. In Expression 2, the integration process is specified, in which the remembering self is defined as an evaluation of remembered experiences. In this expression, *default()* signifies that a default function is applied over the remembered experiences, in order to create a summary of those experiences. What form this function should take exactly is an open question.

To illustrate, Kahneman and Riis (2005) provide an example of a music lover who reports that his whole experience of listening to a long symphony on a record is ruined by a loud noise caused by a scratch in that record towards the end. Evidently, the noise did not affect the experiences lived by the experiencing self before the noise, but rather the summary of the experiences constructed by the remembering self. Applying the expressions to the symphony example, the music lover may not remember every experience during the symphony, as every note can be perceived as an experience (Expression 1). Furthermore, as the music lover is convinced that the negative experience overshadows the rest of their experiences, in this example, function *default()* may signify that only the most intense remembered experience is considered, or that the most intense remembered experience receives a high weight, while the rest of the remembered experiences receives a very low weight (Expression 2).

In the psychological literature, Expressions 1 and 2 (although not formalized as such) have been studied separately. Experimental studies suggest that they may be affected by specific biases and heuristics. Concerning the retrieval of moment-to-moment experiences (Expression 1), Tversky and Kahneman (1973) wrote about the *availability heuristic*. This heuristic entails that when people try to determine the frequency of events, they tend to retrieve examples, which are more easily retrieved when they are salient. Consequently, salient experiences may be regarded as frequently occurring. Relatedly, Bower (1981) first wrote about *mood-state-dependent memory*, the finding that people are better at retrieving memories that are congruent with their current mood. Later reviews and meta-analyzes, although reporting mixed findings, did find support for this phenomenon (Ucros, 1989; Matt et al., 1992; Kihlstrom et al., 1999; Barry et al., 2004; Gaddy and Ingram, 2014), and suggested that depressed patients may especially suffer from its consequences (Matt et al., 1992; Kihlstrom et al., 1999; Barry et al., 2004; Gaddy and Ingram, 2014). In terms of Expression 1, this means that salient experiences (availability heuristic), or experiences that are congruent with current mood (mood-state-dependent memory) have a higher probability of being remembered than experiences that are not salient or congruent with current mood, and therefore, the subset of remembered experiences out of the experiencing self is indeed “*biased*” in the sense that it is an unrepresentative sample of the entire experiencing self.

Concerning the integration of experiences in the remembering self (Expression 2), the psychological literature includes multiple theories about how people summarize their retrieved experiences. Each of these theories essentially posits more or less specific *default()* functions for Expression 2. Fredrickson and Kahneman (1993) describe several strategies that may be employed to summarize experiences. In a strategy they refer to as *temporal integration*, the intensities of experiences are summed over time. That is, when experience intensity would be plotted as a function of time, according to this strategy, a quantification of the overall experience is given by the area under the curve. This implies that an evaluation of the overall experience relies on both the intensity and duration of the experience, and that each new experience should add to the total experience. The *default()* function in Expression 2 would then become an integral:


rememberingself=∫rememberedexperiencestimeframe (3)

Here, both the intensity and the temporal progression of experiences are continuous, within a specified timeframe. Fredrickson and Kahneman (1993) reported results that imply that people generally did not use the temporal integration strategy, as they did not take the duration of an affective episode into account when they evaluated the pleasantness of this episode, a bias Fredrickson and Kahneman labelled *duration neglect*. For example, when people would consider two episodes with equal negative experiences throughout, but a strong difference in length, they would not rate the longer episode as more unpleasant. Duration neglect seemed to increase when more time had passed before overall evaluations were made.

Fredrickson and Kahneman also describe a contrasting strategy: instead of temporal integration, people may employ a strategy of *weighted averaging*, in which they retrospectively ascribe a weight to each experience (not to be confused with the intensity of the experience) that may be equal to zero. In this case, not every new experience needs to add to the total experience, and as a consequence, duration of the experience need not affect the total experience. This function can be expressed as:


$$\begin{array}{l} rememberingself=\\ \frac{\Sigma_{i=1}^{n}weights_{i}\;remembered\;experiences\;time\;frame_{i}}{\Sigma_{i=1}^{n}\;weights_{i}} \end{array}$$ (4)

where *n* is the number of (discrete) remembered experiences within a specified timeframe, *weights* is a vector of weights of the same length that remembered experiences are multiplied by, and *i* is the index of both remembered experiences and weights. We have described an example of this strategy when we applied Expression 2 to the symphony example, in which the music lover gave a particularly high weight to their strongest experience. Some authors suggest that people may use a specific weighted combination when they are asked to provide a general rating of an unpleasant episode, which has been termed the *peak-end rule*. That is, these authors report that in situations where people were asked to retrospectively rate a physically unpleasant experience (i.e., a painful surgery or putting their hand in an ice bath), they tended to take the average of the peak value and the end value of their momentary reports of this experience, rather than the average across the complete time span (Fredrickson and Kahneman, 1993; Kahneman et al., 1993; Redelmeier and Kahneman, 1996). In terms of Expression 4, this means that these authors propose a specific vector of weights, in which both the weight at the index of the strongest remembered experience and the last (remembered) experience should be equal and non-zero while the rest of the weights should be zero. This heuristic in the integration process bares resemblance to the availability and mood-congruency biases described previously for the retrieval process. That is, in the retrieval process, salient experiences may have a higher probability of being remembered, and in the integration process, they may be overrepresented compared to less salient experiences. Similarly, in the retrieval process, experiences that are congruent with current mood have a higher probability of being remembered, while in the integration process, current mood may be overrepresented compared to less recent experiences.

To summarize, the experimental literature suggests that the remembering self does not provide a summary of moment-to-moment experiences that is representative of all moment-to-moment experiences. However, although they may be “*biased*,” evaluations by the remembering self contain important information, as previously acknowledged by other authors (Kahneman and Riis, 2005; Conner and Barrett, 2012). These evaluations are peoples’ way of making a coherent narrative out of their experiences, and they form the basis of peoples’ decision making and long-term planning. For example, they may drive a person to seek help for their psychological problems, determine whether treatment works for them, or to quit treatment. Therefore, we agree with previous authors (Kahneman and Riis, 2005) that in order to fully understand what certain experiences mean to individuals, both their remembering self and experiencing self should be measured. If there is indeed a strong difference between all true moment-to-moment experiences and the true evaluations that people have about those experiences, this may be worthwhile to explore in a personalized feedback procedure. Notably however, these differences, possibly driven by the availability heuristic, mood-congruent memory, or peak-end rule are presumed to occur in healthy individuals. This means that a difference between the remembering self and experiencing self is to be expected for all.

Some authors additionally suggest that these biases may be emphasized by psychopathology (Matt et al., 1992; Kihlstrom et al., 1999; Barry et al., 2004; Gaddy and Ingram, 2014). Such an emphasized bias seems like a precondition for the personalized feedback procedure, as in this case there is a specific difference between moment-to-moment experiences and evaluations of those experiences that people may gain insight into. Specifically, for people suffering from a certain disorder, their remembering self may overrepresent their negative experiences disproportionally compared to healthy people, and it may help them to change this. However, although a comparison between remembering self and experiencing self seems to lie at the core of the personalized feedback procedure, measurements of the remembering self are rarely included in current designs, and measurements of the remembering self concerning the same information as the personalized feedback based on the experiencing self are never gathered; we will discuss this in detail in section “Affecting Evaluations, Using Feedback About Experiences.” Before we can discuss this lack of comparison between measurements of the remembering self and the experiencing self however, we need to address the specific challenges in gathering these measurements of the remembering self and experiencing self outside of the experimental setting.

## Measuring Experiences in Clinical Research

In this section, we will discuss how the remembering self and the experiencing self are measured, and how these measurements are typically used in clinical research. The main assumption that relates to these issues is that retrospective assessment does not represent the experiencing self well as it rather represents the remembering self, while EMA does represent the experiencing self well (see assumption 2 in Table 1). First, we will discuss how the remembering self relates to retrospective assessment. We believe that retrospective assessment can be formalized as a restricted version of the remembering self that participants may or may not be able to produce. Second, we will discuss to what extent the experiencing self is captured by EMA. In EMA, there is a wide variety of flavors that to a varying degree tap into the experiencing self. By discussing how the remembering self and the experiencing self map onto empirical data, we will show how moving from true processes to approximations of these processes comes with specific caveats.

### Retrospective Assessment: Measuring Narratives About Experiences

Traditional instruments for measuring psychopathology, such as clinical interviews and self-report questionnaires, which we will refer to as retrospective assessment, ask participants to retrospectively report on experiences over a prolonged period of time. The relation between the remembering self and retrospective assessment is to the best of our knowledge never made explicit. Let us assume that:


$$\begin{array}{l} Retrospectiveassessment=\\ formulateRA\left(rememberedexperiencestimeframe\right) \end{array}$$ (5)

That is, although retrospective assessment is structurally similar to the remembering self (see Expression 2), retrospective assessment differs from the remembering self in that it requires people to apply a *specific* function over remembered experiences within a timeframe specified by the assessment, whereas the remembering self is free to apply any function over any remembered experiences. This specific function in retrospective assessment is signified by *formulateRA()* in Expression 5, and it may differ from the function *default()* for the remembering self in Expression 2. Combined, the specific function and timeframe (specified in Expression 5 by addition *timeframe*) may result in a difference between retrospective assessment and the remembering self. For example, someone may not be convinced that they meet the conditions for a certain psychological problem (e.g., a depressive episode), which would be their default summary of their remembered experiences by their remembering self. However, using retrospective assessment, it may turn out that they do meet the conditions for this problem. In fact, the goal of function *formulateRA()* is often to find support for diagnostic decisions, based on diagnostic manuals. In practice, the “specific” function *formulateRA()* is often vague, making it unclear what is measured with retrospective assessment exactly.

To illustrate, let us consider the diagnostic process for major depressive disorder, as it is encoded in DSM-5 (American Psychiatric Association, 2013). Specifically, we will look at criterion A for diagnosing major depressive disorder. In order to meet criterion A, someone must report at least five out of nine possible symptoms, at least one of which must be either “*depressed mood*” or “*loss of interest or pleasure*.” These five symptoms need to have been present “*during the same 2-week period*” and represent a “*change from previous functioning*.” In addition, except for the symptom “*recurrent thoughts of death* […], *recurrent suicidal ideation* […], *or a suicide attempt* […],” all symptoms need to have been present “*nearly every day*.” Obtaining such symptom information from participants is an elaborate procedure that is sometimes accomplished by means of structured interviews, carried out by qualified interviewers (Kessler et al., 1994, 2005a,b). In order to answer the questions in such interviews and questionnaires alike, participants are essentially required to construct a summary out of their day-to-day experiences, thoughts, feelings and behaviors. For example, given the structure of typical clinical interviews and questionnaires, in order to answer whether one has suffered from a symptom like *depressed mood*, one would have to answer the following questions:

a. When did I experience *depressed mood* during the 2-week time window I’m being interviewed about?
b. Did I experience *depressed mood* most of the day, nearly every day?

The first question is related to the retrieval process (see Expression 1), and will lead to a set of remembered experiences. The second question is related to the integration process specified by retrospective assessment (see Expression 5), and requires one to sum the length in time of remembered experiences per day and compare this to the total number of wake time in a day. Then, the number of days that the summed experiences cover *most of the day* needs to be counted to reach a decision on whether this was the case *nearly every day*. The fact that the answer should be an approximation, indicates that coming up with a precise solution may not be realistic. This phrasing does leave the opportunity for personal interpretation of when experiences cover *most* of the day, and *nearly* all days. Moreover, participants are sometimes asked to apply a function over a specified period (e.g., 2 weeks for depression, or 6 months for generalized anxiety disorder) that may have happened during the past year, or their entire life (Kessler et al., 1994, 2005a,b). In this case, unless this period was very recent, participants will likely not be able to reproduce questions a and b adequately. That is, the subset of remembered experiences may be too small (i.e., the result of question a) to reliably estimate whether they covered most of the day for most days, during a specific period (i.e., the result of question b) some time ago. As people may not be able to apply the (vague) function *formulateRA()* that is required (see Expression 5), they may instead replace it with an unknown function. We hypothesize that this function is similar to the *default()* function of their remembering self (see Expression 2), because the participant will rely on their default, unrestricted way of processing their experiences. The result may be hard to formalize, and prone to biases described in section “Experiencing and Evaluating Experiences.”

Furthermore, answer-categories of frequently used questionnaires often pertain to a mix of the frequency and severity of symptoms (Beck et al., 1961; Radloff, 1977; Zigmond and Snaith, 1983; Kroenke et al., 2001), for example, answer categories for feelings of sadness in the BDI-II (Beck et al., 1961) feature: “*I feel sad much of the time*” (score 1), and “*I am so sad I cannot stand it*” (score 3). The latter seems like an extra qualification of severity that one may have to consider after evaluating the frequency of feeling sad. Such extra evaluative layers mean that the function that participants need to apply may even vary per answer category.

The previous examples demonstrate that retrospectively answering questions about symptomatology either through traditional interviews or questionnaires often involves many different decisions regarding severity, frequency and aggregation. In other words, the function *formulateRA()* in retrospective assessment is often specified rather vaguely, or is unlikely to be applied precisely, for example because of the specified timeframe that needs to be integrated over. In such cases, we hypothesize that instead, people may use their own function, which would be the *default()* function of their remembering self (see Expression 2), or a mixture of the required *formulateRA()* function and their *default()* function. In terms of Expression 5, it means that function *formulateRA()* that strictly needs to be applied to remembered experiences if instructions were followed can in practice take many different forms. As a result, it is not clear what is measured exactly in retrospective assessment. For the diagnostic process, this may not be a particular problem, as a personal approximation may suffice, although some authors contest this (van Os et al., 2013). However, when the goal is to pinpoint experiences in retrospective assessment, and how they relate to experiences captured by EMA – which we believe lies at the core of studying the personalized feedback procedure – more specific and realistically achievable *formulateRA()* functions may be required, as we will discuss in more detail in the final section of this manuscript.

### EMA: Measuring Moment-to-Moment Experiences

In comparison to the previously described questions in retrospective assessment, questions in EMA are often relatively simple: people repeatedly report on Likert or visual analogue scales to what extent they experience positive affect (e.g., cheerfulness or enthusiasm), negative affect (e.g., sadness and nervousness) or symptoms (e.g., fatigue and concentration problems). Additionally, participants usually report on variables such as their current environment and events that are happening. The high frequency of such measurements, combined with the specified timeframe (e.g., “*how sad do you feel right now*”) indeed seem related to the experiencing self, described previously as living short moments without reflecting on them much. Concerning the data gathering process in EMA, Kahneman and Riis (2005) explicitly, and many others implicitly assume that:


EMA⊂experiencingself (6)

That is, like remembered experiences (see Expression 1), EMA takes an incomplete subset of the experiencing self as it only draws a sample out of all experiences. In contrast to remembered experiences, it is assumed that the EMA subset represents the experiencing self relatively well.

However, there are different variants of EMA, which because of their different timing and content seem to represent the experiencing self to varying extent.

In terms of timing, in a method referred to as the *Experience Sampling Method* (ESM), prompts occur at random moments within fixed time intervals (e.g., waking hours are divided in blocks of 4 h, and within each block, the prompt occurs at a random moment). Some authors have adopted the term “*gold standard*” for ESM (Kahneman et al., 2004; Dockray et al., 2010; Lucas et al., 2021), possibly as it takes a subset out of the experiencing self that seems random to a high extent, although this is not made explicit. Alternatively, in a method referred to as the *diary method*, prompts occur at fixed time intervals (e.g., exactly every 4 h during waking hours). This method is clearly random to a lesser extent, as there is a chance that people are measured only during specific situations and environments throughout their day because of their daily routine. Alternatively, participants can be asked to fill in questionnaires after specific events (De Beurs et al., 1992; Stein and Corte, 2003; Wonderlich et al., 2015), which is referred to as *event-contingent sampling*. In this case, the EMA subset can be considered to be a non-random sample that is complete regarding a specific event, which would also make it a representative subset of the experiencing self.

In terms of the general content of questions, the time window that the questions refer to impacts the extent to which EMA can be conceptualized as a representative subset of the experiencing self. In many designs, people are asked about their experiences in the exact moment which as mentioned earlier, seems closely related to the experiencing self (e.g., “*how sad do you feel right now?*”; c.f., Thomas and Diener, 1990; Parkinson et al., 1995; Barrett, 1997; Ebner-Priemer et al., 2006; Ben-Zeev et al., 2009, 2012; Ben-Zeev and Young, 2010; Dockray et al., 2010; Burns et al., 2011; Bylsma et al., 2011; Wenze et al., 2012; Kramer et al., 2014; van der Krieke et al., 2016; Kroeze et al., 2017; Lay et al., 2017; Colombo et al., 2019, 2020; Neubauer et al., 2020; Lucas et al., 2021; Ornée et al., 2021). An important drawback to this procedure however, is that there is a high chance that salient experiences will be missed. First, there is a small chance for a measurement occasion to occur exactly during the salient experience. Second, there may be a higher probability of non-response during salient experiences, resulting in a missing observation. For example, a person suffering from panic attacks will not likely fill in a questionnaire when experiencing a panic attack. On the positive end of the experiential continuum, while being on a fun date, a person may also not be inclined to fill in a questionnaire. Such missing-not-at-random (MNAR) data are a potential problem of EMA that is rarely addressed. This MNAR problem implies that where retrospective assessment may overemphasize salient experiences, EMA may underemphasize them.

These drawbacks of EMA can be alleviated by asking participants about experiences since the last measurement occasion (c.f., Ben-Zeev et al., 2012; Fernandez et al., 2017; van Roekel et al., 2017; Fisher et al., 2019; Rinner et al., 2019) or since some time interval (c.f., Gloster et al., 2008; Solhan et al., 2009; Priebe et al., 2013; Torous et al., 2015; Schuler et al., 2021). In this approach, participants are essentially required to recall and aggregate over some limited time interval. As these features are the key features in retrospective assessment (see Expression 5), a more appropriate term for this procedure may be *Ecological Retrospective Assessment* (ERA). Furthermore, as the specific function that needs to be applied to aggregate the experiences since the last occasion is typically not specified (e.g., “*how sad did you feel since the last measurement occasion?*”), this form of assessment seems rather close to the remembering self, which is also free in applying a summary function. In a special case of the diary method, referred to as the *daily diary method*, participants only report about their experiences at the end of the day (c.f., Thomas and Diener, 1990; Parkinson et al., 1995; Kardum and Daskijević, 2001; Kranzler et al., 2004; Simpson et al., 2011; Naragon-Gainey et al., 2012; Wonderlich et al., 2015; Krenek et al., 2016; Campbell et al., 2017; Urban et al., 2018; Neubauer et al., 2020; Mneimne et al., 2021). A related method is the *Day Reconstruction Method* (DRM) proposed by Kahneman et al. (2004); (c.f., Miron-Shatz et al., 2009; Dockray et al., 2010; Bylsma et al., 2011; Kim et al., 2013; Tadić et al., 2014; Lucas et al., 2021). In this method, participants are asked to divide their previous day in episodes, and report how they felt during each of these episodes. This procedure results in a more specific subset of experiences than the daily diary method; however, it still requires aggregation of experiences within episodes. In the remainder of this manuscript, we will refer to a procedure in which participants are asked to report on about experiences in the exact moment as EMA, while we will refer to any data collection procedure in which participants retrospectively assess their moment-to-moment experiences sequentially as ERA.

**Figure 1:**
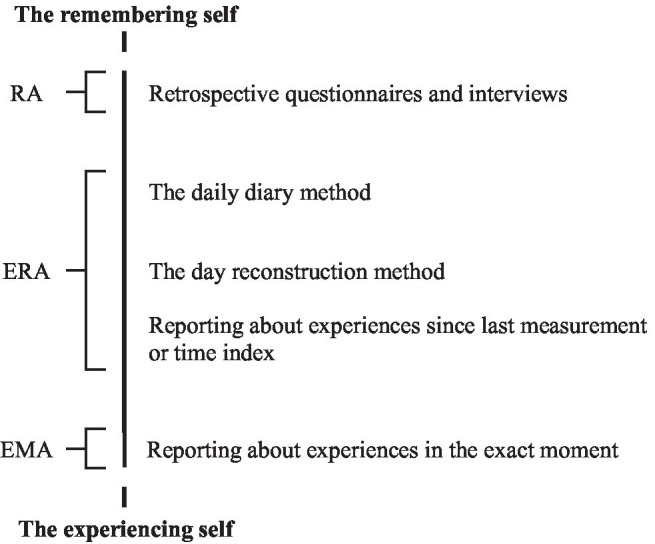
Different data collection procedures on the continuum from the RS to the ES. RA, Retrospective Assessment, in which participants need to summarize remembered experiences over a prolonged period. ERA, Ecological Retrospective Assessment, in which participants need to sequentially summarize remembered experiences over a shorter period. EMA, Ecological Momentary Assessment, in which participants report on their current experiences sequentially.

The previous reflections demonstrate that EMA and retrospective assessment are not two clearly distinct categories. As we have described in the previous subsections, retrospective assessment does not map onto the remembering self completely and EMA maps onto the experiencing self to varying extent. Furthermore, even when participants are inquired to report on experiences in the exact moment during EMA, it may not be completely non-evaluative. Specifically, when asked about experiences in the exact moment, they may question why they feel the way they do, the answer to which may refer to something that is not taking place in the current moment. Emotions can be conceptualized as responses to emotional events (Kuppens and Verduyn, 2015), and many symptoms are actually oriented towards other constructs (Borsboom et al., 2019). For example, a socially anxious person will be anxious about giving a presentation, which is another construct, occurring outside of the moment that EMA is about. Or the depressed person may feel worthless, because they did not do anything all day. More fundamentally, in order to come up with a meaningful quantification of how one is feeling right now, one will have to compare one’s experiences to some previous experiences. As such, it may be more appropriate to think about the data collection procedures we discuss in this section as lying on a continuum from remembering to experiencing (see Figure 1), as some authors have suggested earlier (Neubauer et al., 2020). Note that the DRM takes an especially debatable position on the continuum from the experiencing self to the remembering self. That is, as it is only applied once and not necessarily in participants own environment, it bares strong resemblance to retrospective assessment in its procedure, while in terms of content, various authors claim that it should be close to the experiencing self. Future studies may help determine the orientation of the DRM on the continuum pictured in Figure 1.

To summarize the reflections in this section, in addition to true differences between the experiencing self and the remembering self, there may be different influences of data collection procedures. The assumption that retrospective assessment, in its current form, seems to reflect the remembering self rather than the experiencing self seems justified. Retrospective assessment can be conceptualized as a specific form of the remembering self that imposes a function on remembered experiences dictated by the formulation in the assessment. This function is often vague or may not be realistically achievable, and as a result, people may apply their own unknown function that may indeed be close to the default function of their remembering self. However, the assumption that EMA represents the experiencing self well seems to be oversimplified. That is, different data collection procedures that are traditionally considered part of EMA have to balance the possibility to capture salient experiences with the introduction of retrospectivity. As a result, it seems that different data collection procedures may lie on a continuum from the remembering self to the experiencing self. Comparisons between different data collection procedures with an emphasis on either the remembering self or the experiencing self may highlight where the difference between experiences and retrospective evaluations of those experiences is the strongest. Such comparisons are fundamental for the personalized feedback procedure: if the goal is to educate people about their true moment-to-moment experiences using personalized feedback, we need to make sure that we capture these moment-to-moment experiences adequately. Specifically, we need to establish which differences may be true differences between the experiencing self and the remembering self, and which may be due to the applied data collection procedure. In the next section, we will discuss studies that have compared different retrospective assessment, ERA and EMA data collection procedures.

## A Difference Between Measurement Types?

In the previous section, we have discussed how data collected through different data collection procedures may lie on a continuum from the experiencing self to the remembering self, and how differences between these types of data may come with specific interpretations. In this section, we will discuss studies that have actually compared these different types of data. The main assumption with regard to the personalized feedback procedure is that data from different data collection procedures should not align (see assumption 3 in Table 1). This idea seems to stem from main assumption 1, which is that there is a difference between the experiencing self and the remembering self. Below, we will evaluate this (mis)alignment, discuss how differences between these types of data are generally interpreted, and how we would interpret them using the previously described considerations.

In order to compare retrospective assessment data to EMA or ERA data, a function needs to be applied to EMA/ERA data to reduce them to the same number of observations as in retrospective assessment (typically one). The same holds when ERA is compared to EMA if ERA contains fewer observations than EMA. Taking these aggregation procedures into account, we can specify comparisons between retrospective assessment, ERA and EMA in the following expressions:


$$\begin{array}{l} formulateRA\left(rememberedexperiencestimeframe\right)\neq \\ aggregateEMA\left(EMA\right) \end{array}$$ (7)

$$\begin{array}{l} aggregateERA(formulateERA(remembered\\ experiencestimeframeshort))\neq \\ aggregateEMA(EMA) \end{array}$$ (8)

$$\begin{array}{l} formulateRA\left(rememberedexperiencestimeframe\right)\neq \\ aggregateERA(formulateERA(remembered\\ experiencestimeframeshort)) \end{array}$$ (9)

Here, functions *formulateRA()* and *formulateERA()* need to be applied by participants to remembered experiences during retrospective assessment and ERA, respectively. The functions *aggregateEMA()* and *aggregateERA()* are used during analysis to reduce the number of observations in EMA or ERA, respectively, to the same number as the number of observations that they are compared to. Importantly, aggregate functions as well as formulate functions differ not only per category of assessment (i.e., EMA, ERA, and RA), but on study level. Across studies however it is generally expected that these different summaries of different types of data will not align perfectly, which is signified by the ≠ sign. Although these expressions are specified at the level of the individual, in practice they are typically studied on a group level, using a (hierarchical) regression framework to express a difference. In the sub-sections below, we will address these comparisons separately for each expression.

### Retrospective Assessment vs. EMA

When retrospective assessment is compared to EMA (Expression 7), measures that are close to the remembering self are essentially compared to measures that are close to the experiencing self (see Figure 1). In such comparisons in the current literature, functions *formulateRA()* and *aggregateEMA()* are typically different. For example, in most studies comparing RA to EMA, participants are asked to apply a *general rating* during retrospective assessment (i.e., function *formulateRA()*; e.g., “*how sad did you feel last week*”), and this general rating is compared to the (weighted) mean (i.e., function *aggregateEMA()*) of their EMA data. Note that when participants are asked for the mean of their experiences explicitly during retrospective assessment (c.f., Kardum and Daskijević, 2001), functions *formulateRA()* and *aggregateEMA()* would be equal.

For affective measures, it is generally reported that retrospective assessment scores are higher than the mean that is observed in EMA data (Ebner-Priemer et al., 2006; Ben-Zeev et al., 2009; Wenze et al., 2012) although in some studies this effect is reported only for positive affect (Parkinson et al., 1995) negative affect (Colombo et al., 2020), or not at all (Barrett, 1997). This difference between retrospective assessment and aggregated EMA has been labelled the *memory experience gap*. Specific memory experience gaps have been reported for depression symptoms in healthy and depressed participants (Ben-Zeev and Young, 2010). Some authors have found that retrospective assessment scores for positive affect were lower than aggregated EMA scores for participants suffering from depression symptoms (Colombo et al., 2019, 2020), or borderline personality disorder (Ebner-Priemer et al., 2006). Some authors report that healthy participants showed specifically higher retrospective assessment scores than aggregated EMA scores for positive affect compared to depressed or anxious participants (Ben-Zeev et al., 2009; Wenze et al., 2012; Colombo et al., 2019, 2020).

The memory experience gap is generally interpreted as evidence for bias of the remembering self as compared to the experiencing self. However, as described in section “Measuring Experiences in Clinical Research,” some differences between retrospective assessment and EMA could also be due to underrepresentation of salient experiences in EMA. Furthermore, the fact that the mean is chosen by default to aggregate EMA measurements may be problematic. That is, suppose that someone perfectly reports on the mode of their affective experience during retrospective assessment as their general rating, yet this mode is compared to the mean of EMA measurements. It seems unjust to then suppose that the difference between the two types of scores is due to bias at the level of retrospective assessment; the scores are simply about different summary measures. This problem can be obviated by specifically asking for the mean during retrospective assessment. As a trade-off, such measurements may stray from the remembering self, as they are more specific, and therefore constrained.

### Ecological Retrospective Assessment vs. Ecological Momentary Assessment

In comparison studies of ERA and EMA (Expression 8), some authors that employ a daily diary sampling strategy seem to consider this form of ERA a measurement of the remembering self (Parkinson et al., 1995). Other authors that use the DRM as ERA seem to suggest that both this form of ERA and EMA measure the experiencing self (Dockray et al., 2010; Bylsma et al., 2011; Kim et al., 2013; Lucas et al., 2021). These seemingly conflicting interpretations may be unified by acknowledging that there is a grey area in the middle of the continuum from the experiencing self to the remembering self, as we have described earlier (see Figure 1). Furthermore, similar to the comparisons of EMA and retrospective assessment (see Expression 7), for comparisons of EMA to ERA data, functions *formulateERA()*, *aggregateEMA()*, and *aggregateERA()* (see Expression 8) are typically not equivalent. For example, when daily diary scores are compared to EMA scores within the same day (Parkinson et al., 1995; Neubauer et al., 2020), function *formulateERA()* may signify that a general rating is required during daily diary assessment. Function *aggregateEMA()* would signify that EMA scores are aggregated within days, for example using the mean. Finally, in this example, function *aggregateERA()* would signify that the daily diary scores are not summarized, for example by multiplying each score by 1. In such a comparison, Parkinson et al. (1995) found that daily diary scores were higher than aggregated EMA-scores for positive affect only, while Neubauer et al. (2020) found a stable difference for negative affect, and conflicting results for positive affect.

When the DRM is compared to EMA, function *formulateERA()* signifies that a general rating should be given to each specified episode in the DRM. A comparison of means may then entail that functions *aggregateERA()* and *aggregateEMA()* should take the mean of DRM and EMA scores, respectively. Mixed results are reported for comparisons of means of DRM and EMA scores (Dockray et al., 2010; Bylsma et al., 2011; Kim et al., 2013; Lucas et al., 2021). Notably, in studies that include measures of within-person alignment between matched DRM and EMA-scores in addition to group-level alignment in means (Kim et al., 2013; Lucas et al., 2021), within-person alignment between matched DRM and EMA scores was quite low.

When ERA is considered a procedure that is close to the experiencing self on the continuum from the experiencing self to remembering self (see Figure 1), studies in which a difference between ERA and EMA is reported (i.e., daily diary studies and within-person DRM studies) could imply that there is a difference in how these data collection procedures capture the experiencing self. When ERA is considered a procedure that is close to the remembering self, these differences could be indicative of a difference between the remembering self and the experiencing self. However, in these comparisons as well, bias in EMA or a difference between summary functions *formulateERA()* and *aggregateEMA()* may influence the results. Taken together, these results are in line with the notion that ERA may take up a position in the middle of the continuum from the experiencing self and the remembering self. That is, results of daily diary to EMA comparisons vary in their conclusions about a difference between ERA and EMA for either positive or negative affect, and results of DRM to EMA comparisons are even more diffuse. The latter also implies that when people are asked to apply a more specific function *formulateERA()* to their remembered experiences during ERA, as is the case in the DRM procedure, the difference in scores compared to EMA is less clear – that is, no consistently higher scores for DRM – than when function *formulateERA()* is more general, as is the case in retrospective assessment and daily diary methods. More specifically, this implies that the DRM does not favor salient experiences as much through its function and/or the subset of invoked remembered experiences, as do retrospective assessment and ERA featuring general ratings.

### Retrospective Assessment vs. ERA

When retrospective assessment is compared to ERA (Expression 9), some authors seem to consider ERA a measurement of the experiencing self, as they claim to study a memory-experience gap (Miron-Shatz et al., 2009; Tadić et al., 2014; Urban et al., 2018; Rinner et al., 2019). However, another valid interpretation could be that in this comparison, measurements that are rather close to the remembering self (i.e., ERA) are essentially compared to measurements that are even closer to the remembering self, as they involve a longer period in time (i.e., RA; see Figure 1). As such, we believe that any difference between these measures may also indicate that the remembering self changes over time, rather than that the remembering self and the experiencing self diverge. In all studies that we encountered except Thomas and Diener (1990) and Solhan et al. (2009), functions *formulateRA()* and *formulateERA()* are equal for this comparison (see Expression 9). For example, *formulateERA()* may signify that during ERA, participants need to fill in how often they had an experience since the last measurement occasion. Function *formulateRA()* may then signify that during retrospective assessment, participants fill in how often they had an experience during the entire period. Finally, function *aggregateERA()* may signify that the sum of ERA measurements should be considered. In these studies, it is generally reported that on group-level, retrospective assessment scores for affective experiences are higher than ERA scores (Thomas and Diener, 1990; Parkinson et al., 1995; Kardum and Daskijević, 2001; Miron-Shatz et al., 2009; Tadić et al., 2014; Urban et al., 2018; Rinner et al., 2019). When ERA would be considered a measurement of the experiencing self, these findings may indicate that there is a difference between the experiencing self and the remembering self. However, as one may also argue that both measures are rather close to the remembering self, these findings can also be interpreted as support for the notion that when people need to integrate over a longer period of time, biases towards salient experiences are more likely to take effect.

In addition to affective experiences, a comparison between ERA and retrospective assessment is typically used in the symptom domain, likely because ERA is better able to capture salient experiences in comparison to EMA. For symptoms, the results are much more diffuse, with some authors reporting higher retrospective assessment scores for panic attacks (De Beurs et al., 1992), alcohol abuse (Simpson et al., 2011), psychotic experiences (Ben-Zeev et al., 2012), PTSD symptoms (Campbell et al., 2017; Schuler et al., 2021), and social phobia (Rinner et al., 2019), while others have found relatively good congruence for OCD (Gloster et al., 2008) and PTSD (Naragon-Gainey et al., 2012) symptomatology. Mixed results have been reported for bulimia nervosa (Stein and Corte, 2003; Wonderlich et al., 2015) and borderline personality disorder (Solhan et al., 2009; Mneimne et al., 2021) symptoms. Effects in the opposite direction (i.e., lower retrospective assessment scores) have also been reported for PTSD (Priebe et al., 2013), depression (Torous et al., 2015), and alcohol use disorder symptomatology (Kranzler et al., 2004; Krenek et al., 2016). Although further research is required to reach a final conclusion, these results indicate that specific forms of psychopathology may to a varying degree show a difference between ERA and retrospective assessment.

To summarize, these comparisons between EMA, ERA, and retrospective assessment do not provide unambiguous conclusions about a difference in scores of different data collection procedures, let alone a difference between the remembering self and the experiencing self. For affective data, results appear to be more similar than for symptom data, as they generally suggest that retrospective assessment scores for both positive and negative affect are higher than EMA and ERA scores, and that daily diary scores are higher than EMA scores. However, for the comparison of affective data using DRM and EMA, results are more scattered. For comparisons of symptom data, which are typically made using ERA and retrospective assessment, results vary strongly. This may partly be due to the characteristics of symptoms that are compared, such as the timing (e.g., prolonged vs. instantaneous) and nature (e.g., inherently salient vs. hardly noticeable). However, varying results are also reported for the same symptoms. In addition to differences due to the data collection procedures and the type of experiences they try to capture, some suggest that a difference in scores is influenced by individual characteristics other than psychopathology (Barrett, 1997; Dockray et al., 2010; Naragon-Gainey et al., 2012; Krenek et al., 2016; Campbell et al., 2017; Lay et al., 2017; Urban et al., 2018; Neubauer et al., 2020; Lucas et al., 2021). In line with this reasoning, various authors report relatively high inter-individual variability in the difference in scores between assessment methods (Miron-Shatz et al., 2009; Ben-Zeev et al., 2012; Tadić et al., 2014; Krenek et al., 2016; Neubauer et al., 2020). Finally, these differences could also be explained by design choices other than the data collection procedure and included experiences, such as the studied timeframe (ranging from 1 to 90 days), and the analysis strategy (e.g., simple aggregation vs. hierarchical models; matched vs. non-matched DRM). Sample sizes also varied strongly. Future studies should quantify the extent to which these design choices impact the results of these comparisons, for example using meta-analysis.

The comparison of retrospective assessment data to EMA/ERA data lies at the core of the personalized feedback procedure, as a difference between the two indicates that there is the opportunity to gain insight. Therefore, it is essential to investigate what we may expect in terms of general trends for certain groups of people, and possible artefacts under different methodological circumstances. From the previous discussion it seems as though this investigation is far from over. Notably, in terms of interpretation, ERA appears to be a grey area that is considered by some to measure the experiencing self, while others consider it to measure the remembering self. Acknowledging this grey area, while making the contrast between measurements closer to the experiencing self or remembering self explicit, for example using the expressions that we use throughout this manuscript, may help interpreting differences that are found. All in all, it seems as if any difference between different types of data should be interpreted with great care.

## Affecting Evaluations, Using Feedback About Experiences

In the previous section, we have described how the comparison between different sources of data comes with many different interpretations, only some of which point in the direction of a difference between the remembering self and the experiencing self. In this section, we will describe how a potential difference between the remembering self and the experiencing self is instrumentalized in the personalized feedback procedure.

Essentially, the personalized feedback procedure uses a presumably relevant summary of a presumably representative subset of the experiencing self in order to promote insight into psychological functioning. Any change in insight that this summary may bring about, can be considered part of the remembering self, which is after all a persons’ evaluation of their experiences. Therefore, the goal in the personalized feedback procedure is to reshape the remembering self so that it is somehow more reflective of a relevant summary of the experiencing self. Along these lines, insight can then be quantified as the extent to which the remembering self is similar to the relevant summary of the experiencing self, such that high similarity between the remembering self and a summary of experiencing self means a high degree of insight. The final assumption that we will evaluate is that an increase in insight can be achieved through the personalized feedback procedure (see assumption 4 in Table 1). These concepts are somewhat harder to capture in a mathematical expression, but may be formalized as:


$$\begin{array}{l} feedback\left(EMA\;or\;ERA\right)\to \\ default\left(rememberedexperiences\;pre\right)\approx \\ ideal\left(rememberedexperiencespost\right) \end{array}$$ (10)

Here, function *feedback()* signifies the summary function that is applied to EMA or ERA data for the personalized feedback. These EMA or ERA data are considered a representative subset of the experiencing self, and the summary function *feedback()* that is applied to these data is considered particularly relevant. The arrow signifies that *feedback(EMA or ERA)* (i.e., the personalized feedback) influences the unrestricted *default()* function applied to remembered experiences before seeing the feedback which is signified by addition *pre* (together, these form the remembering self). Finally, the ≈ sign expresses that *default(remembered experiences pre)* should become more similar to an *ideal()* summary function applied to a set of remembered experiences that may be different from the subset of remembered experiences before seeing the feedback, which is signified by the addition *post*. Function *feedback()* is similar to function *ideal()* that it should promote. For example, if function *feedback()* is to take the mean, researchers hope to achieve that participants ascribe approximately equal weights to their remembered experiences, which would be function *ideal()*. In practice, it is generally not made explicit what function *ideal()* that the feedback should help establish should be. Note that *feedback(EMA or ERA)* is on the level of data, while all other elements in this expression describe a change in a true underlying process, that needs to be translated to data. We will return to expressions featuring only elements on the level of data (which makes them testable), later in this section. To illustrate Expression 10, suppose that someone is convinced that they are happy when they play video games, because they have some particular positive memories of playing these games. This would be their *default(remembered experiences pre)* (i.e., their remembering self). However, after seeing that compared to other activities, their mean positive affect while playing games based on EMA or ERA is rather low [i.e., *feedback(EMA or ERA)*], they may re-evaluate. First, they may remember other, less positive experiences than before, which would alter their set of remembered experiences so that it is closer to their experiencing self (i.e., the difference between remembered experiences *pre* and *post*). Second, they may choose to integrate these experiences into their final evaluation, which may alter their *default()* function so that it becomes more similar to function *ideal()*, in which remembered experiences receive approximately equal weight. However, before this person decides to alter their remembering self [i.e., *default(remembered experiences pre)*], they must be convinced that the EMA or ERA dataset is representative of their experiencing self, and that function *feedback()* is a relevant summary function. In section “Measuring Experiences in Clinical Research,” we have discussed caveats for the former requirement, most importantly in this regard that EMA data may underemphasize salient experiences. In the next sub-sections, we discuss considerations for whether function *feedback()* is relevant.

### Current Applications of Feedback

We will continue by describing the types of *feedback()* functions that are generally applied to EMA or ERA data in the personalized feedback procedure. As discussed in the previous section, in the literature that compares different data types, the mean is typically used as a function to summarize EMA or ERA data. In the personalized feedback literature, previous research has reported on the effects of providing participants with personalized feedback about their mean levels of (positive) affect in their daily lives, and the relative time that participants spent doing certain activities (Burns et al., 2011; Kramer et al., 2014; Hartmann et al., 2015; Simons et al., 2015; Snippe et al., 2016; van Roekel et al., 2017; Ornée et al., 2021).

However, summaries of EMA or ERA data that are used for personalized feedback can also be more complex than taking the frequency of occurrence (as in activities) or the mean (as in affect). Frequently applied methods aim at providing patients with feedback about the associations between experiences. These analyses, which are sometimes referred to as *network* analyses, treat EMA/ERA data as *timeseries* data, and provide information about the *temporal associations* between moment-to-moment experiences in terms of (auto)-correlation coefficients. For example, such analysis of EMA/ERA data may result in the finding that for a person, the experience of feeling anxious at one point in time predicts the experience of feeling down at a later point in time (Bringmann et al., 2013). Some authors have suggested that these types of network models may help guide interventions (Fisher and Boswell, 2016; Borsboom, 2017; Fernandez et al., 2017; Fisher et al., 2019; Hofmann and Hayes, 2019),^2^ and the first empirical efforts that have provided personalized feedback based on network analyzes have started to arise (van der Krieke et al., 2016; Kroeze et al., 2017; van Roekel et al., 2017; Epskamp et al., 2018). Kroeze et al. (2017), for example, analyzed the network of a patient suffering from treatment resistant anxious and depressive symptoms. For this patient, *physical discomfort* (operationalized as heart pondering, sweating, trembling) turned out to be related to many other variables in the network. Discussing this finding with the patient led her to trying out interoceptive exposure, a form of therapy that she had been previously reluctant about.

Taken together, all personalized feedback applications based on EMA data, either based on descriptive statistics or associations between variables, indeed provide patients with an EMA data-driven summary, and some report on whether this receiving this feedback was associated with a positive outcome. However, none of these procedures currently include any measurement of the remembering self to compare the personalized feedback to. As such, any possible increase in insight is currently often left in the open. Specifically, in many cases, the personalized feedback procedure lacks both a pre- and a post-measure of insight (c.f., Burns et al., 2011; Kramer et al., 2014; Hartmann et al., 2015; Simons et al., 2015; Snippe et al., 2016; van der Krieke et al., 2016; van Roekel et al., 2017; Ornée et al., 2021). Instead, in some of these studies, alleviation of symptoms is used as a post-measure (Kramer et al., 2014; Hartmann et al., 2015; Simons et al., 2015; Snippe et al., 2016; van Roekel et al., 2017). As such, it is assumed in these studies that the increase in insight described in Expression 10 should have either directly led to symptom alleviation, or should have led to a change in some mechanism, that in turn resulted in symptom alleviation. Earlier proposed mechanisms are increased positive affect (Hartmann et al., 2015), feelings of empowerment (Simons et al., 2015), or change in activities (Snippe et al., 2016). However, although changes in these presumed mechanisms have been documented, any change in insight that may have preceded a change in these mechanisms has not been recorded in these studies. This makes it hard to ascribe the change in symptom alleviation to gained insight from the personalized feedback, rather than other aspects of the procedure.

In other cases, a post-measure is included in the form of a qualitative description about the general impression of the personalized feedback procedure, after it has taken place (Kroeze et al., 2017; Epskamp et al., 2018). In such cases, a qualitative pre-measure of insight would make clearer to what extent the feedback brought about any change in insight, or whether it was actually rather confirmation of something that could have been extracted from the participant before seeing the feedback using appropriate retrospective assessment. For example, previous authors have suggested that the process of filling in EMA questionnaires may by itself promote emotional awareness (Kauer et al., 2012; Widdershoven et al., 2019). When an increase in awareness has taken place before the feedback phase, the difference between pre-measure and the provided feedback may be small.

### Measuring the Impact of Personalized Feedback

Notably, pre-measures of insight are not considered in any of the studies that we have described above. We propose that the procedure of gathering a pre- and a post-measure gives an indication of the extent to which Expression 10 holds. The procedure of gathering a pre-and a post-measure allows for a more confirmatory/deductive process, in which hypotheses by the patient are tested with data, and the data-based evidence is evaluated afterwards. This stands in contrast to the highly explorative/inductive process that the personalized feedback procedure is currently. If we apply our proposed deductive process to the previous video game example, after a period of EMA or ERA data collection, the person receiving the feedback would first be asked to estimate the mean of their positive affect while gaming; this would be their pre-measure. Afterwards, they would be confronted with their personalized feedback, in the form of their mean positive affect based on their EMA or ERA data, along with their mean positive affect during other activities. Finally, they would be asked again what they think their mean positive affect should be while gaming, which would be their post-measure. If their post-measure resembles the personalized feedback while their pre-measure did not, this may be interpreted as an increase in insight. In this proposed deductive procedure, there are actually four of such general outcomes that we have defined in the following expressions:


$$\begin{array}{l} preFeedback\left(remembered\;experiences\;pre\right)\neq \\ postFeedback\left(rememberedexperiencespost\right)\&\\ postFeedback\left(rememberedexperiencespost\right)\approx \\ feedback\left(EMA\;or\;EMA\right) \end{array}$$ (11)

$$\begin{array}{l} preFeedback\left(rememberedexperiences\;pre\right)\approx \\ postFeedback\left(rememberedexperiencespost\right)\&\\ postFeedback\left(rememberedexperiencespost\right)\approx \\ feedback\left(EMA\;or\;EMA\right) \end{array}$$ (12)

$$\begin{array}{l} preFeedback\left(rememberedexperiences\;pre\right)\approx \\ postFeedback\left(rememberedexperiencespost\right)\&\\ postFeedback\left(rememberedexperiencespost\right)\neq \\ feedback\left(EMA\;or\;EMA\right) \end{array}$$ (13)

$$\begin{array}{l} preFeedback\left(rememberedexperiences\;pre\right)\neq \\ postFeedback\left(rememberedexperiencespost\right)\&\\ postFeedback\left(rememberedexperiencespost\right)\neq \\ feedback\left(EMA\;or\;EMA\right) \end{array}$$ (14)

Here, function *feedback()* is again the function that is selected for the personalized feedback. Functions *preFeedback()* and *postFeedback()* in these expressions signify that the function that participants are asked to apply during pre- and post-measures, respectively, are approximations of this function. For example, participants will most likely not calculate (auto)correlation-coefficients in their mind, but may think about predictive relations. These functions as well as the subsets that these functions are applied to may be different between pre- and post-measures, as described in the previous video game example in which the participant may remember certain experiences after seeing the feedback (see Expression 10). This is signified by additions *pre* and *post*. Expression 11 describes a scenario that is congruent with the previous video game example. That is, the pre-measure and personalized feedback do not align, and the person decides that the personalized feedback may be correct. As a result, their post-measure will resemble their personalized feedback. Note that only in this case expressed in Expression 11 there would be increased insight. Expression 12 describes a scenario in which pre-measure and personalized feedback do align. In this scenario, the person undergoing the procedure can find confirmation of their initial evaluation and their post-measure will resemble both pre-measure and personalized feedback. Expression 13 describes a more problematic scenario. Similar to Expression 11, pre-measure and personalized feedback do not align. However, in the scenario described by expression 11, the person decides not to believe the personalized feedback, and to stick to their initial evaluation expressed in their pre-measure. This poses a problem as in this case either the person or the personalized feedback is wrong (or both), but we do not know which. Finally, Expression 14 also describes a scenario in which the participant is skeptical towards the personalized feedback. In this scenario, pre- and post-measures do not align, and the post-measure and personalized feedback also do not align. This may for example occur when the post-measure falls in between pre-measure and the feedback, or is similar to the pre-measure is some aspects, and similar to the feedback on others. However, it could also theoretically be that the post-measure is very dissimilar to both feedback and pre-measure.

Although problematic in terms of interpretation, Expressions 13 and 14, which are indicative of skepsis towards the personalized feedback, may in a clinical setting spark a meaningful discussion between clinical researcher/clinician and person undergoing the personalized feedback procedure. Based on such a conversation, they may for example start a new personalized feedback procedure, possibly involving a different data collection procedure or a different *feedback()* function to extract the relevant information from the available EMA/ERA data. For research on population-level differences, trends in skepticism may indicate either a difference between the experiencing self and the remembering self, or a methodological problem that would be interesting to look into.

That is, as we have described in the previous sections, in addition to true differences between the experiencing self and the remembering self, the personalized feedback report may be affected by the data collection procedure it was based on. Furthermore, a serious consideration for personalized feedback is that the *feedback()* function is to some extent arbitrary, and that it depends on a number of methodological choices. The latter was demonstrated by Bastiaansen et al. (2020) who asked 12 research teams to analyze the same EMA dataset, and found that the selected targets for intervention differed strongly. Interestingly, several research teams that analyzed the dataset suggested that the person undergoing the procedure should be involved in the process, which is also what we recommend in our proposed deductive process.

To summarize, adding a pre- and a post-measure of insight may safeguard against overinterpretation of a relatively arbitrary summary of possibly ambiguous data. As it involves more specificity in the measurement of relevant constructs, and a more explicit evaluation of hypotheses, this ultimately seems like a more scientific approach. In the following, we will summarize our conclusions about the current position of personalized feedback research based on Expressions 1–4, and provide a roadmap with steps to push personalized feedback research forward.

## Discussion and Future Research

From sections “Experiencing and Evaluating Experiences to Affecting Evaluations, Using Feedback About Experiences,” we have formalized assumptions in the personalized feedback procedure in expressions, and discussed empirical evidence for these assumptions. We have done so using a bottom-up approach. That is, we started with the assumptions in theories about true experiences and evaluations of those experiences. Then we discussed how these theories are assumed to be translated to specific types of data, and what we may expect to find when these different types of data are compared. Finally, we discussed how it is assumed that providing people with a summary of their moment-to-moment experiences may promote insight into their psychological functioning. In this section, we will summarize our conclusions regarding the underlying assumptions using a top-down approach, starting at the personalized feedback procedure, and moving back to the core theories about experiences and evaluations of those experiences.

In general, our discussion of expressions has made clear that summary functions and the subsets they are applied to are often unknown, and deserve further exploration. In the following, we will propose leads for such explorations at each discussed assumption. Before we do so, it is important to make clear that the theoretical constructs, the expressions that are used to define those constructs, and the lines of research that are proposed to study them in this manuscript are not exhaustive, and may be subject to debate. Sparking such a debate is the overarching goal of this manuscript. That is, we believe that explicitly addressing these issues will move the scientific field of personalized feedback based on EMA/ERA forward.

### The Impact of Personalized Feedback

The main assumption in the actual procedure of providing people with personalized feedback is that a data-driven summary based on moment-to-moment experiences can affect retrospective evaluations of those experiences (see main assumption 4 in Table 1). However, whether this is indeed the case is not yet investigated, as the procedure currently does not include the required outcome measures to establish an increase in insight. As such, there is no empirical support for this assumption. In terms of Expression 10, it is often not made clear in current applications what the properties should be of the *ideal()* function that a participant should ideally apply to a representative subset of experiences, and it is not recorded whether their *default()* function applied to their default subset of remembered experiences changes towards this *ideal()* function, applied to a different subset of remembered experiences. We have suggested that pre- and post-measures should be included in order to effectively study this claim. Next, we will provide suggestions for how such measures can be constructed for the various types of personalized feedback.

**Figure 2:**
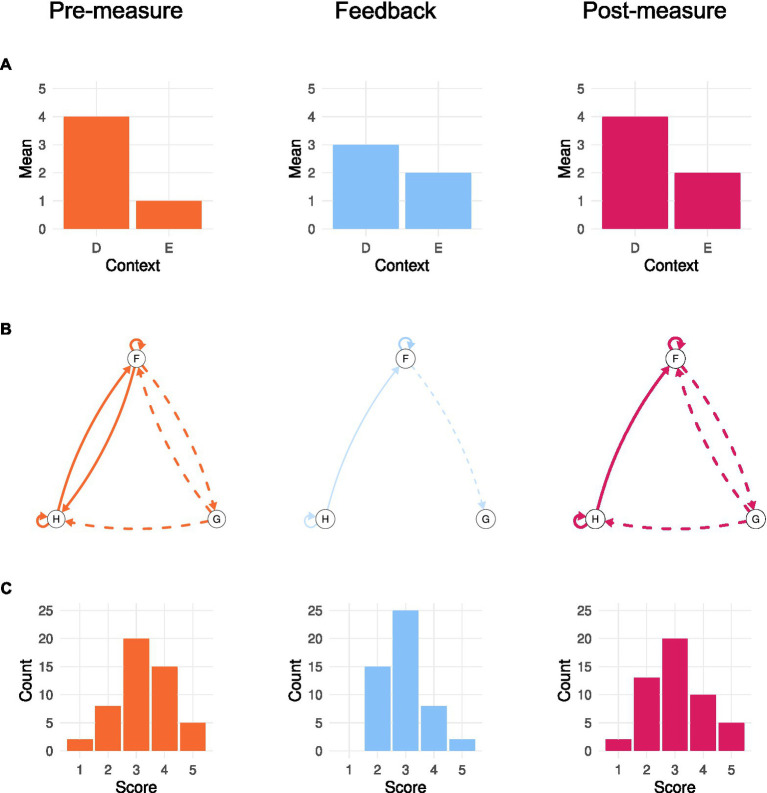
Fictious examples of the proposed deductive feedback process of collecting a pre-measure (column 1), providing data-driven feedback (column 2), and collecting a post-measure (column 3). Row **A** shows hypothetical outcomes for a comparison of means of an experience (*y*-axis) in different contexts *D* and *E* (*x*-axis). Row **B** shows hypothetical outcomes of a comparison of predictive relations between experiences *F*, *G*, and *H*. Row **C** shows hypothetical outcomes for a comparison of distributions/histograms of scores (*x*-axis) for an experience.

For personalized feedback about the mean of certain experiences in different contexts, the deductive procedure seems relatively straightforward, as this statistic can be explained to participants with relative ease. After such an explanation, participants would estimate the mean of their experiences (in different contexts). Then, they would see the mean of each experience (in each context) based on EMA/ERA. Finally, participants would indicate what they think should be the mean (in each context) after seeing the feedback (see Figure 2A).

For pre- and post-measures about relations between experiences, new challenges arise. That is, it seems unlikely that the average person will be able to come up with a reasonable estimation of their temporal (auto)correlation coefficient without extensive help or simplification. A relatively strongly simplified method may be to let people estimate whether relations between experiences over time should be either positive, negative or absent (see Figure 2B). A more elaborate method that can account for differences in predictive strength of relations between experiences, could be an extension of the so-called *perceived causal relations* (Frewen et al., 2012, 2013). In the current application of this method, participants rate on a 0–10 scale whether they think that each included variable *caused* all other included variables. In order to make a network based on these perceived causal relations comparable to a network based on EMA-data, some adjustments will have to be made. To start, the questions may have to be reformulated so that they resemble the predictive relationships that the network based on EMA-data features. Instead of asking to what extent variables caused other variables, participants may for example be asked to what extent they believe that when they had a certain experience, this generally was associated with having this experience or other experiences at some later point in time. Given this slight change in formulation, it may also be easier for participants to estimate the predictive influences that variables may have on themselves, which is currently not done in the perceived-causal-relations method. These so-called *self-loops* reflect the earlier described inertia of experiences, and should be featured in a network of what we may call *perceived predictive relations* in order to make it comparable to a network based on EMA data. Importantly, even with these adjustments it is not clear-cut how these perceived predictive relations map onto the auto-correlation coefficients that are typically used for estimating personalized networks. For example, these coefficients may change depending on the included variables, and the specific time-interval between measurement occasions. Making sure that participants take such effects into account in their estimations may be particularly challenging.

In general, a big challenge for these methods will be that participants should understand the functions *preFeedback()*, *feedback()* and *postFeedback()* (see Expressions 11–14) that they need to apply to their remembered experiences. As such, it is very important to evaluate the extent to which participants understood the procedure. Until more data have been gathered on this topic, the outcome of the described deductive process seems like our best option to establish validity of the personalized feedback. In other words, as a true model (i.e., summary function) and true experiences (i.e., the experiencing self) will always be unknown, we would rather rely on an evaluation of both EMA and retrospective assessment data by the person that these data are about, than on the EMA data alone. In clinical contexts, a discussion of the EMA/ERA-data-driven feedback between patient and clinician may already happen qualitatively to some extent, but it would be scientific to also quantify it in terms of a pre- and a post-measure. Furthermore, it would be relevant for the field to document and publish both these qualitative and quantitative explorations of insight.

Some may challenge that increasing insight is an explicit goal of their personalized feedback. We urge those to make their proposed goal of personalized feedback explicit, as well as the presumed mechanisms that should lead to this goal. Others may argue that we do not need to know the specific mechanisms under the hood of personalized feedback procedure, as it has already proven its utility in terms of positive outcomes in some studies. However, in addition to the scientific goal of expanding knowledge, we believe that studying these mechanisms may help in making the procedure more efficient. As such, there is much to gain on both a scientific and practical level.

Taking the reaction of participants to the feedback seriously seems relevant as selecting a *feedback()* function (see Expressions 10–14) does not appear to be a clear-cut, generic procedure, especially when the summary becomes more complex. The field of idiographic (network) analysis of psychological data is relatively young, its methodologies are in constant development, and are subject of debate (c.f., Kuiper and Ryan, 2018; Bringmann et al., 2019; Dejonckheere et al., 2019; Schuurman and Hamaker, 2019; Dablander et al., 2020; Bringmann, 2021). As such, validation by the person that these summaries are about seems like an especially interesting venue to explore.

### The Comparison of Different Measurement Types

Although no quantitative pre- and post-measures of insight have been gathered yet in a personalized feedback setting, the literature about group-level differences in scores of different data collection procedures may in theory give us a preliminary indication of what we may expect for the comparison of a pre-measure to the personalized feedback on a nomothetic level. There is however an important difference between current comparison studies and our proposed deductive process. Specifically, in current applications of retrospective assessment and ERA participants are typically asked for a general rating in *formulateRA()* and *formulateERA()* (i.e., “*how bored did you feel?*”; see Expressions 5, 7, and 9), while the proposed deductive procedure in personalized feedback uses a more specific function for *preFeedback()* and *postFeedback()* (e.g., “*how bored did you feel on average?*”; see Expressions 11–14). By making this function more specific, participants may be less likely to apply their *default()* function (see Expression 2). In turn, using a more specific summary function on a set of remembered experiences during the pre-measure stage may already promote function *ideal()* by itself. For example, as noted before, applying the average to experiences may promote another way of processing remembered experiences, in which they are weighted more equally.

If the results of current comparisons of different data collection procedures were to give an indication of what we may expect in the personalized feedback procedure, this indication would not be clear-cut. Results are not unified, and are possibly influenced by a multitude of methodological decisions. Although within this field, it is generally assumed that a difference between data sources is indicative of a difference between moment-to-moment experiences and evaluations of these experiences, further study of the data collection procedures is required in order to disentangle true differences between the experiencing self and remembering self from methodological artefacts. As such, we believe that main assumption 3, which is that data from EMA, ERA and retrospective assessment will not align because of a difference between the remembering self and the experiencing self (see Table 1), deserves further exploration. Meta-analysis seems like a suitable instrument to explore the source(s) of these differences. Further research into group-level differences may also highlight what we can expect in terms of general differences between different types of data for certain groups, particularly what can be considered a “*healthy*” difference. For example, many empirical studies report that healthy participants retrospectively estimate their positive and negative affect to be higher than the mean of their EMA data (Thomas and Diener, 1990; Ebner-Priemer et al., 2006; Ben-Zeev et al., 2009; Wenze et al., 2012; Lay et al., 2017; Urban et al., 2018; Colombo et al., 2019, 2020; Rinner et al., 2019). Some authors even propose that healthy functioning is associated with a particularly pronounced difference between data sources for positive affect (Ben-Zeev et al., 2009, 2012; Wenze et al., 2012; Colombo et al., 2019, 2020; Rinner et al., 2019), which may either be indicative of a remembering self that is biased or a particular MNAR problem in EMA for positive affective experiences in healthy individuals.

If there is indeed a “*healthy bias*” in which retrospective evaluations of positive affect are stronger than moment-to-moment experiences, this may mean that adjusting peoples’ evaluations to align with a summary of their EMA may not suffice. In other words, the goal in this case may not be to promote insight based on a representative summary of the experiencing self, but rather to establish a specific positive bias. Realistic personalized feedback may not be a suitable instrument to achieve this. Instead, people may for example be encouraged to specifically focus on positive experiences, as is common in cognitive therapy (Beck, 1979). The “*healthy bias*” problem exemplifies that formal theories about the goals of personalized feedback, and the roads towards those goals are needed.

### Different Measurement Types

The other explanation for the finding that retrospective assessment scores are found to be higher than EMA scores for positive affect in healthy individuals forms a general consideration for EMA data. Namely, that EMA (i.e., assessment about experiences in the exact moment) may underrepresent salient experiences. Because of this possible MNAR problem, the common assumption that EMA takes a representative subset of the experiencing self, while retrospective assessment does not (see main assumption 2 in Table 1), deserves further exploration. When studying this phenomenon, we run into a similar problem that we run into when the participant is skeptical towards their personalized feedback. That is, the true set of experiences will be unknown, and our best bet for empirical data is to make a comparison to peoples’ personal evaluations of their experiences, which are inherently incomplete and possibly biased.

As mentioned earlier, a venue to explore in order to minimize retrospective bias is to make the function *formulationRA()* (see Expressions 2, 7, and 9) more specific and similar to function *aggregateEMA()* or *aggregateERA()*. The easiest example is to explicitly ask for the mean of an experience during RA when this mean is to be used to summarize the EMA data. A more elaborate specific function would be to let participants estimate distributions retrospectively, for example with a method called the trial roulette method (Gore, 1987; Morris et al., 2014; Veen et al., 2017). This novel form of retrospective assessment is an example of promising new approaches for obtaining more realistically achievable summaries of experiences that are both specific and result in a high similarity between functions *formulateRA()* and *aggregateEMA()*/*aggregateERA*. An application of this procedure to the personalized feedback procedure can be found in Figure 2C. Results for such comparisons are however yet to be documented.

Another method for studying the causes for a difference between scores of different data collection procedures is through (Monte Carlo) simulations (Haslbeck et al., 2019). In such simulations, the true experiential and retrospective processes, as well as the measurement processes of these true processes can be simulated given some theoretical properties (e.g., the MNAR problem). The difference between simulated measurements would then be compared to differences in empirical data, to see if the specified theoretical property can account for the empirical differences.

Finally, a related field of research is the comparison of ERA/EMA and RA measurements to psychophysiological measurements of a concurrent period (c.f., Adams et al., 2017). In order to validate that psychophysiological measurements are associated with certain experiences, EMA measurements are currently used. However, including ERA/RA to this comparison may lead to new insights about moments that were particularly salient, and may reduce bias due to missing data.

### Experience and Retrospective Evaluation

With regard to explicit measurements of the remembering self, a venue to explore is to do (qualitative) studies about decision making in retrospective assessment and ERA, in order to capture the default strategies that people employ when they are asked to provide a general rating of their experiences. As a qualitative measure, participants may for example be asked how they arrived at their final conclusion of their general rating of experiences in functions *formulateRA()* or *formulateERA()* (see Expressions 2, 7–9). Such meta-measurements may teach us about the possibly various strategies that people use to make their retrospective summaries.

Quantitative decision-making studies, like the early experiments by Kahneman and colleagues also still seem relevant. Such experimental studies may complement ecologically valid studies by pinpointing decision processes. That is, in these experimental designs, the experiential trajectory can be controlled, and as such an approximation of a true experiential process can be achieved. This facilitates falsification, as we saw in the case of temporal integration as a *default()* function of the remembering self (Fredrickson and Kahneman, 1993).

Together, these suggested explorations about the impact of personalized feedback, meta-analyzes on differences between data collection procedures, simulation studies about the mechanisms behind a difference between retrospective assessment and EMA/ERA, and qualitative and experimental decision making studies may serve as instruments in answering the core theoretical question of whether and how our true experiences are substantially different from our retrospective evaluations of those experiences, which brings us back to main assumption 1 (see Table 1). We believe that studying this assumption in the domain of real-life psychological phenomena can be impactful for the personalized feedback procedure, as well as many other fields in psychology. With this manuscript, we hope to contribute to a discussion of the state of the assumptions that form the foundation of this type of research.

## Author Contributions

IL, AC, and NS were involved in the conceptualization of the manuscript. IL, AC, JV, and NS contributed to the original draft of the manuscript. IL and NS established the applied methodology. IL did the investigation and validation. IL, JV, and NS contributed to the final edited version of the manuscript. All authors contributed to the article and approved the submitted version.

## Conflict of Interest

The authors declare that the research was conducted in the absence of any commercial or financial relationships that could be construed as a potential conflict of interest.

## Publisher’s Note

All claims expressed in this article are solely those of the authors and do not necessarily represent those of their affiliated organizations, or those of the publisher, the editors and the reviewers. Any product that may be evaluated in this article, or claim that may be made by its manufacturer, is not guaranteed or endorsed by the publisher.

## Footnotes

^1^EMA measurements fall into two broad categories: (psycho)physiological (e.g., heart-rate, skin-conductance) and self-report (e.g., affective- and psychopathological experiences). For physiological measurements, the related field of biofeedback aims at making people more aware of their (psycho)physiological experiences.

^2^Note that these proposed interventions typically do not involve a phase of providing feedback to the participant. Instead, they are directly selected based on the network models.
